# Coupling effects on photoluminescence of exciton states in asymmetric quantum dot molecules

**DOI:** 10.1186/1556-276X-9-297

**Published:** 2014-06-12

**Authors:** Nelson R Fino, Angela S Camacho, Hanz Y Ramírez

**Affiliations:** 1 Departamento de Física, Universidad de los Andes, Bogotá D.C. 111711, Colombia; 2 Departamento de Física, Universidad Antonio Nariño (UAN), Bogotá D.C. 111511, Colombia; 3 Grupo de Física Teórica y Computacional, Escuela de Física, Universidad Pedagógica y Tecnológica de Colombia (UPTC), Tunja 150003, Colombia

**Keywords:** Quantum dot molecules, Photoluminescence, Excitons, Diamagnetism

## Abstract

We present a theoretical study of photoluminescence from exciton states in InAs/GaAs asymmetric dot pairs, where interdot coupling is reached via magnetic field in the Faraday configuration. Electronic structure is obtained by finite element calculations, and Coulomb effects are included using a perturbative approach. According to our simulated spectra, bright excited states may become optically accessible at low temperatures in hybridization regimes where intermixing with the ground state is achieved. Our results show effective magnetic control on the energy, polarization and intensity of emitted light, and suggest these coupled nanostructures as relevant candidates for implementation of quantum optoelectronic devices.

## Review

### Introduction

The development of novel devices for spintronics and quantum information processing (e.g., single-photon emitters and quantum logic gates) has been a primary motivation in the development of nanostructured semiconductors in the last years. Confined excitons offer the possibility of using laser for initialization, readout, and coherent manipulation of spins. InAs quantum dots (QDs) may be fabricated by molecular beam epitaxial deposition on GaAs, in which lattice mismatch leads to the formation of InAs clusters through a process known as Stranski-Krastanov growth [1].

When this method is repeated in upper layers, obtention of stacked structures is favored. It is important to note that even if the different layers are growth under very similar conditions, strain leads to non-identical dots, and slight differences in their energy levels are unavoidable [2].

In analogy with well-known phenomena in molecule formation, coupling between ‘artificial atoms’ in a stacked pair should be tunable via the geometry parameters (static coherent tuning) or by applying external fields (dynamic coherent tuning) [3,4]. Spectroscopic signatures of coupling in charged quantum dot molecules were directly observed several years ago by Krenner et al. [2] and Stinaff et al. [5]. Nevertheless, how controllable this coupling might be and the role of Coulomb interactions in such a tunability are still subject of investigation.

The most usual mechanism to couple dots is the application of an electric bias field [6,7]; however, this involves reduction of the oscillator strength due to induced decrease of the electron-hole overlap, so presenting an unavoidable inconvenience for optical work with excitons. That is not an issue in the case of magnetic field-driven coupling.

In this paper, we study the photoluminescence spectrum (PL) of an asymmetric quantum dot pair (AQDP). To do it, we proceed as follows: In the first part, we model the stacked double-dot structure and calculate the ground state energy for the electron and hole in each of the involved dots. Then, to describe the field-dot interaction, we apply the Fermi golden rule to the AQDP states. At the final part, we simulate the PL spectrum and comment on the obtained results.

### System model

**Figure 1:**
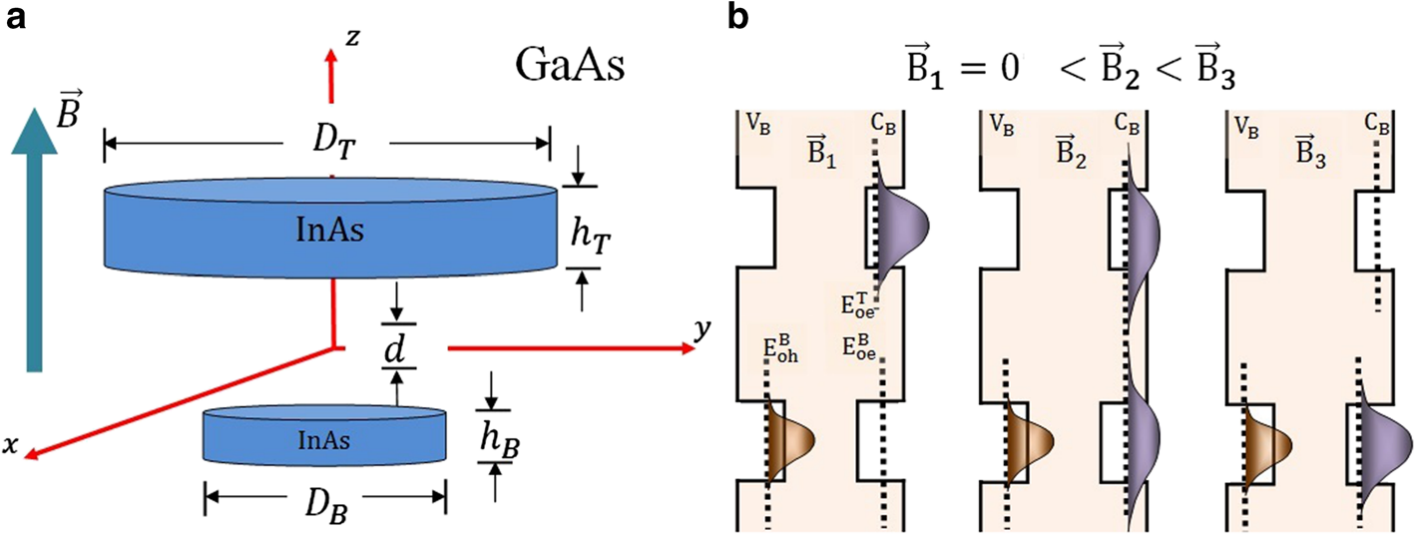
**Asymmetric quantum dot pair and band structure. (a)** Schematics of the asymmetric quantum dot pair. **(b)** Depiction of the band structure illustrating the changes on the eigenstates induced by the magnetic field.

The system under study is an AQDP, which is composed of two InAs quantum dots embedded in a matrix of GaAs. The dots are disks aligned in the *z* direction, ensuring cylindrical symmetry (see Figure 1). The energy levels are tuned via magnetic field, which is applied in the growth direction of the structure (Faraday configuration). There are two important effects of the field on the system: the Zeeman splitting which is due to the opposite spin projections^a^[8], and the diamagnetic shift that reflects increase of the spatial confinement [3,9-12].

To calculate the energy ground state for electron and hole, depending on external magnetic field, we use the Ben Daniel-Duke equation:

(1) $$H=\frac{1}{2}{\vec{\Pi }}_{r}\cdot \frac{1}{m}{\vec{\Pi }}_{r}+V(\vec{r}),$$

where ${\vec{\Pi }}_{r}=-\mathrm{i\hbar}\nabla_{r}+\text{eA}(\vec{r})$ is the electron (hole) momentum operator, ∇_*r*_ is the spatial gradient, $A(\vec{r})$ is the potential vector that in this case is chosen of the form $A=\frac{B}{2}(-y\hat{ı}+x\hat{ȷ})$, to describe a field in the growth direction, *m* is the effective mass of electron (hole), and $V(\vec{r})$ is the confinement potential. In the present work, to solve this eigenvalue equation, we use the finite element method (FE) by means of the software Comsol (Comsol, Inc., Burlington, MA, USA)^b^[13]. We consider AQDPs charged with one electron and one hole (neutral exciton *X*^0^). Then, we choose for the *X*^0^ basis $\left(\begin{array}{ll} 0 & ↑\\ ⇓ & 0 \end{array}\right)$, $\left(\begin{array}{ll} 0 & ↓\\ ⇑ & 0 \end{array}\right)$, $\left(\begin{array}{ll} ↑ & 0\\ ⇓ & 0 \end{array}\right)$and $\left(\begin{array}{ll} ↓ & 0\\ ⇑ & 0 \end{array}\right)$. In this basis, the first (second) row refers to electron (hole), and the first (second) column refers to the bottom (top) dot, the single arrow (double) refers to electron spin projection $\pm \frac{1}{2}$ (heavy-hole pseudospin projection $\pm \frac{3}{2}$). Implicitly, in this basis, there are two kinds of excitons: *direct exciton* when electron and hole are in the same dot, and *indirect exciton* when they are in different dots.

In such a basis, excitons have total angular momentum ±1 (*↓⇑* and *↑⇓*), meaning, they are optically active (can be coupled to photons). With all these considerations, the *X*^0^ Hamiltonian matrix is

(2) $$\begin{array}{c} H=\left(\begin{array}{cccc} E_{g}+E_{\text{oe}}^{T}+E_{\text{oh}}^{B} & 0 & -t_{e} & 0\\ +Z_{e}+Z_{h}\\ 0 & E_{g}+E_{\text{oe}}^{T}+E_{\text{oh}}^{B} & 0 & -t_{e}\\ & -Z_{e}-Z_{h}\\ -t_{e} & 0 & E_{g}+E_{\text{oe}}^{B}+E_{\text{oh}}^{B} & 0\\ & & -V_{e-h}^{B}\\ & & +Z_{e}+Z_{h}\\ 0 & -t_{e} & 0 & E_{g}+E_{\text{oe}}^{B}+E_{\text{oh}}^{B}\\ & & & -V_{e-h}^{B}\\ & & & -Z_{e}-Z_{h} \end{array}\right),\; \end{array}$$

where *E*_g_ is the energy gap, $E_{\text{oe}}^{B}$ ($E_{\text{oe}}^{T}$) is the ground state energy of the electron on the bottom (top) dot, $E_{\text{oh}}^{B}$ is the ground state energy of the hole on the bottom dot (in the Hamiltonian, this energy appears in all diagonal terms because the hole does not tunnel in the studied field window)^c^[14], *Z*_e_ (*Z*_h_) is the Zeeman splitting of electron (hole), $V_{e-h}^{B}$ is the Coulomb interaction between electron and hole in the bottom dot, and *t*_e_ is the tunnel energy of the coupling interaction which conserves spin orientation. In this Hamiltonian, the Coulomb interaction for the indirect exciton is neglected since it is at least 1 order of magnitude smaller than in the direct exciton case.

### Photoluminescence simulation

In the following, we suppose exciton population generated by non-resonant optical excitation on the AQDP. Thus, we use the Fermi golden rule to calculate the PL spectra of *X*^0^ states in AQDPs. Accordingly, the transition rate *Γ*, from the initial state |*i*> to the final state | *f*>, is given by

(3) $$\Gamma =\frac{2\pi }{\hbar }|>f|H^{\text{int}}|i>{|}^{2}\rho (E),$$

where *H*^int^ means the interaction responsible for the transition, and *ρ*(*E*) is the density of energy states. For each frequency value, the intensity of the signal has to be directly proportional to the total probability of all possible transitions. Hence, the PL intensity is given by

(4) $$I(\omega )\propto \underset{\text{if}}{\sum }|>X_{f}|H|X_{i}>{|}^{2}\rho (E),$$

where |*X*_*i*_> (|*X*_*f*_>) means the initial (final) exciton state with energy $E_{X_{i}}$ ($E_{X_{f}}$). In the case of $X^{0^{\prime }}s$ confined in AQDPs, a photon emission is equivalent to a electron-hole recombination, i.e., single-exciton annihilation. Under this assumption, the final state is the exciton vacuum state |0>. Thus, ensuring energy conservation and considering the 0D nature of the system,

(5) $$I(\omega )\propto \underset{i}{\sum }F(E_{i},T)|>0|H|X_{i}>{|}^{2}\delta (E_{i}-\hbar \omega ),$$

where $F(E_{i},T)=e^{\frac{-E_{i}}{k_{B}T}}/\sum_{i}\frac{-E_{i}}{k_{B}T}$ is the temperature-dependent probability of occupation of state |*i*>, *k*_B_ is the Boltzmann constant, and *T* is the temperature [15]. Using the electron (hole) creation operator over the vacuum state ($c_{j,\sigma }^{†}h_{n,\chi }^{†}|0>$), we can obtain the basis exciton states |*X*_*j*,*σ*,*n*,*χ*_>, which are composed of an electron in the confined stated *j* and spin |*σ*>, and a hole in the confined state *n* and pseudospin |*χ*>. The *X*_i_ states are superpositions of these basis states whose coefficients are obtained by diagonalizing the Hamiltonian in Equation 2. For each of the circular polarization + or -, the interaction can be described by the polarization operator

(6) $$P_{+}^{E}=\underset{n,j}{\sum }S_{n,j}h_{\mathrm{n⇑}}c_{\mathrm{j↓}}$$

(7) $$P_{-}^{E}=\underset{n,j}{\sum }S_{n,j}h_{\mathrm{n⇓}}c_{\mathrm{j↑}}$$

where *S*_*n* *j*_ is the overlap between the envelope part of the electron and hole wave functions in the states |*n*> and |*j*> of the conduction and valence bands, respectively.

## Results and discussion

In the following, we use specific (and realistic) values for the size and confinement offset of the dots. While this apparently implies loss of generality for our results, actually, it allows us to illustrate vividly the impact of size and magnetic field on the emission features of AQDPs.

Although in a dot pair, the relative energy spacing could also be generated and controlled by changes in stoichiometry, bias fields (which would affect significantly the Coulomb interaction), and mechanical stress, among others. Size difference represents the most relevant parameter given the current limitations to obtain dots of identical dimensions. Since all others can be suppressed or strongly minimized at will, we focus on this aspect’s influence.

In the first place, when the diameter of the dot increases, the ground state energy of electron decreases, but its response to the field is larger, i.e., the change of the energy with respect to the field ($\frac{\text{dE}}{\text{dB}}$) grows significantly. For instance, if the diameter of the dot is increased from 15 to 30 nm (height constant of 4.2 nm), the ground state energy decreases in 40 meV at *B*=0, but the energy growth rate in the second case is 2.13 meV/T against 1 meV/T of the first one. Taking this behavior into account, an energy branch corresponding to larger dots starts as the lowest in energy (at *B*=0). It will reach an excited energy branch corresponding to smaller dots at some non-zero field, allowing artificial molecular states. We use this property to determine the dimensions (height and diameter) that permit the indirect exciton branch (the first two states of basis) to start slightly below in energy than the direct exciton branch (the last two states of basis) and then to reach it in a field smaller than 30 T.

Another important quantity, which also depends on the dot size is the Coulomb interaction energy ($V_{e-h}^{B}$) [16-18]. For example, if the diameter of the dot increases from 15 to 30 nm, that energy changes from 19 to 10 meV. These values are small compared to the exciton energy, but are determining for resonant regions.

Thus, we choose two particular AQDPs (one of which exhibits molecular states, while the other one does not) to simulate their corresponding photoluminescence spectra. They allow, by contrast, to observe the very important effects of size and Coulomb interaction to give rise to the appearance of hybridized states. To select the dimensions of the two studied systems, after calculating exciton energies as a function of diameters and heights at *B*=0, we pick a couple of representative AQDP configurations. A interdot distance of *d*=7.8 nm is used in both cases.

**Figure 2:**
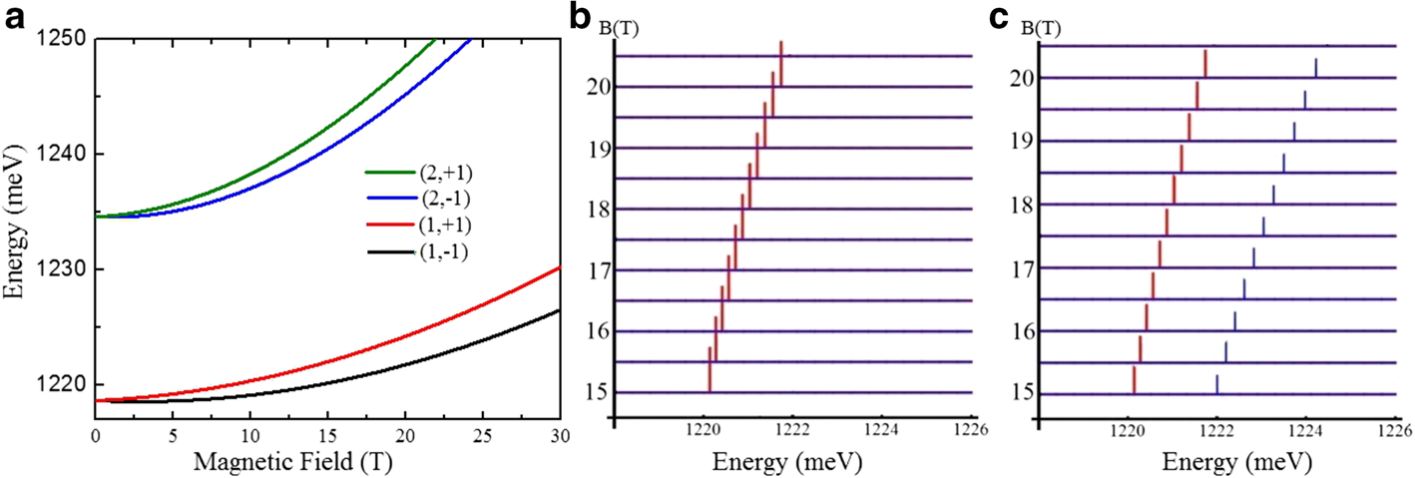
**Dependence of the energy levels and PL spectra of AQDP #1. (a)** Dependence of the energy levels on the magnetic field (the first (second) number in the label indicates the branch (polarization)). **(b)** PL spectrum of an AQDP consisting of a bottom dot with diameter (height) *D*_B_=12 nm (*h*_B_=2.4 nm) and top dot with diameter (height) *D*_T_=24 nm (*h*_T_=1.8 nm) at 5 K. **(c)** As in **(b)** but at 70 K. The red (blue) line corresponds to polarization -1 (+1) in *z*.

First, we study an AQDP (#1) consisting of a bottom dot with diameter (height) *D*_B_=12 nm (*h*_B_=2.4 nm) and a top dot with diameter (height) *D*_T_=24 nm (*h*_T_=1.8 nm). For this configuration, the simulated spectra are shown in Figure 2. In this case, it is clear that the dots do not couple (Figure 2a). In fact, at *B*=0, the energy branch corresponding to indirect states starts above the one corresponding to the direct states, and given the faster growth with field of the first one, the direct branch can not reach the indirect one.

**Figure 3:**
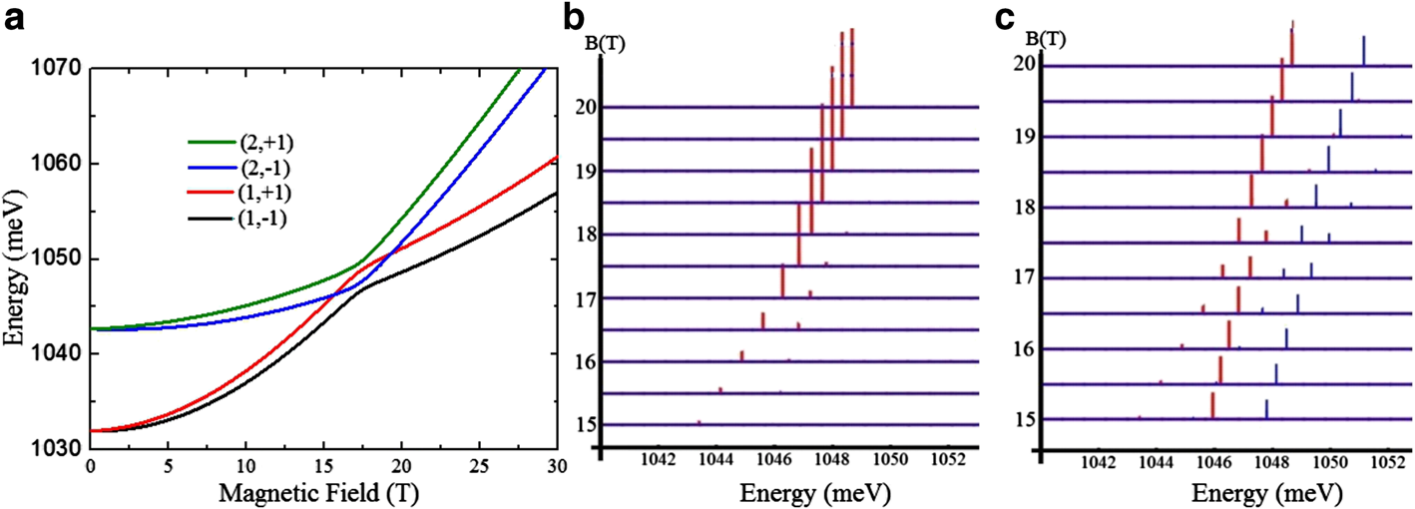
**Dependence of energy levels and PL spectra of AQDP #2. (a)** Dependence of the energy levels on the magnetic field (the first (second) number in the label indicates the branch (polarization)). **(b)** PL spectrum of AQDP consisting of a bottom dot with diameter (height) *D*_B_=15 nm (*h*_B_=4.8 nm) and a top dot with diameter (height) *D*_T_=30 nm (*h*_T_=4.2 nm) at 5 K. **(c)** As in **(b)** but at 70 K. The red (blue) line corresponds to polarization -1 (+1) in *z*.

Increasing the size of the dots (AQDP #2), both of the single-particle ground state energy and the Coulomb interaction decrease. For example, if the bottom dot has a diameter (height) of *D*_B_=15 nm (*h*_B_=4.8 nm) and the top dot has diameter (height) of *D*_T_=30 nm (*h*_T_=4.2 nm) at *B*=0, the energy of the indirect ground state changes from 1,234 to 1,031 meV and that of the direct state changes from 1,238 to 1,042 meV^d^. In this second configuration, the Coulomb interaction is too weak to push the direct branch below the indirect one ($V_{e-h}^{B}$ changes from ∼19 to ∼16 meV). The signal of coupling is observed in this case (Figure 3), especially for the higher temperature, in form of anticrossed states in the PL spectra. This feature is consistent with the experimental observations as reported in [2] and [5], in which interdot coupling is reached via electric field. Such anticrossings (observed in the region 15 T - 20 T), evidence hybridization between the states $\left(\begin{array}{ll} 0 & ↑\\ ⇓ & 0 \end{array}\right)$and $\left(\begin{array}{ll} ↑ & 0\\ ⇓ & 0 \end{array}\right)$which have polarization −1 (red), and between the states $\left(\begin{array}{ll} 0 & ↓\\ ⇑ & 0 \end{array}\right)$ and $\left(\begin{array}{ll} ↓ & 0\\ ⇑ & 0 \end{array}\right)$ with polarization +1 (blue). Via this interdot coupling, energy levels beyond the ground state become optically accessible at reasonably low temperatures (70 K, Figure 3b). This is because the tunneling coupling magnitude is noticeably lower than the typical energy difference between the ground and excited states in single dots. It is worth noting that undesirable thermally driven charge leaking will reduce the PL signal from the dot pair. However, in this case, because coupling is achieved, the energy difference between excited and ground states is much smaller than that between the excited state and the conduction band edge at the hybridization region. Thus, the charge leaking effects on exciton emission from the ground and excited levels are similar, and the PL qualitative features are not expected to change substantially.

## Conclusions

We simulated the photoluminescence spectra of vertically grown pairs of quantum dots and observed that their size is a crucial factor to achieve coupling via magnetic field. Two sets of dots were examined: the first one does not couple because its dimensions strengthen Coulomb interaction and disfavors diamagnetic shift. In contrast, the second one with larger dimensions exhibits a very different behavior as the magnetic field increases, showing the characteristic anticrossings of molecular coupling. The presence of coupling is highly affected by the Coulomb interaction, regardless of the fact that its value is around 2 orders of magnitude smaller than the exciton energy.

Moderate-low temperature (below the nitrogen boiling point) was found enough to optically observe excited states, which is directly related to the small gap between hybridized states in the resonance region. From these results, we conclude that magnetically tuned tunneling coupling eases optical observation of excited states as compared to single-dot states. Furthermore, effective control on the energy, polarization, and intensity of emitted light, through externally applied magnetic field, has been shown which suggests that this type of on-demand coupled nanostructures is a relevant candidate for the implementation of quantum optoelectronic devices.

## Endnotes

^a^ For the electron (hole) *g* factor, we used −0.745 (−1.4).

^b^ The following parameters were used in the calculations: InAs (GaAs) eletron mass 0.023 *m*_*e*_ (0.067 *m*_*e*_), InAs (GaAs) hole mass 0.34 *m*_*e*_ (0.34 *m*_*e*_), and InAs (GaAs) confinement potential *V*_0_=474 meV (258 meV).

^c^ Although the top dot is larger than the bottom one, because of its heaviness, the hole has similar eigenenergies in each of them, and vertical strain effects (as reported in [14]) are likely to be more relevant than those of size. Thus, we assume the ground hole state to remain in the bottom dot.

^d^ An interband gap of 800 meV was used in our calculations.

## Competing interests

The authors declare that they have no competing interests.

## Authors’ contributions

NRF carried out the numerical calculations and drafted most of the manuscript. ASC participated in the design of the study, analysis of results, and contributed to the manuscript. HYR proposed the physical model, oversaw the numerical calculations, and edited the manuscript to its final form. All authors read and approved the final manuscript.

## Authors’ information

NRF is a MSc degree holder and is a lecturer in the Physics Department of UAN. ASC is a Ph.D. degree holder and is a Senior Researcher and Professor in Universidad de Los Andes. HYR is a Ph.D. degree holder and is an Assistant Professor in the School of Physics of UPTC.
